# Hypertension Among Medical Students in Lubumbashi, Democratic Republic of the Congo: Prevalence, Associated Factors and Comparison of the 2025 ACC/AHA and JNC 7 Classifications

**DOI:** 10.1155/ijhy/7252722

**Published:** 2026-09-30

**Authors:** Jacques Mbaz Musung, Emmanuel Kiyana Muyumba, Phillipe Bianga Katchunga, Placide Kambola Kakoma, Yves Idi Isango, Marcellin Bugeme, Berthe Mwad Kon Kamalo, Orly Muyumba Ngungwa, Harvey Kabulo Kapya, Didier Lez Malamba, Olivier Mukuku

**Affiliations:** ^1^ Hypertension/Cardiology Unit Department of Internal Medicine Faculty of Medicine University of Lubumbashi Lubumbashi Democratic Republic of the Congo unilu.ac.cd; ^2^ Gastroenterology Unit Department of Internal Medicine Faculty of Medicine University of Lubumbashi Lubumbashi Democratic Republic of the Congo unilu.ac.cd; ^3^ Cardiology Unit Department of Internal Medicine Official University of Bukavu Bukavu Democratic Republic of the Congo; ^4^ Department of Gynecology-Obstetrics Faculty of Medicine University of Lubumbashi Lubumbashi Democratic Republic of the Congo unilu.ac.cd; ^5^ Department of Neurology Faculty of Medicine University of Lubumbashi Lubumbashi Democratic Republic of the Congo unilu.ac.cd; ^6^ Endocrinology Unit Department of Internal Medicine Faculty of Medicine University of Lubumbashi Lubumbashi Democratic Republic of the Congo unilu.ac.cd; ^7^ Research Department Institut Supérieur des Techniques Médicales de Lubumbashi Lubumbashi Democratic Republic of the Congo istmlubumbashi.net

**Keywords:** ACC/AHA 2025, associated factors, Democratic Republic of the Congo, hypertension, JNC 7, medical students, prevalence, young adults

## Abstract

**Background:**

Hypertension is increasingly recognised among young adults, but its estimated prevalence varies substantially according to the diagnostic criteria used. This study assessed the prevalence and factors associated with hypertension among medical students in Lubumbashi, Democratic Republic of the Congo (DRC), using the 2025 American College of Cardiology/American Heart Association (ACC/AHA) criteria, and compared the findings with the JNC 7 classification.

**Methods:**

We conducted a cross‐sectional study among students aged ≥ 18 years at the Faculty of Medicine, University of Lubumbashi, from July to August 2023. Blood pressure and anthropometric measurements were obtained using standardised procedures, and sociodemographic, behavioural and family‐history data were collected using a structured questionnaire. Hypertension was defined according to the 2025 ACC/AHA criteria and, for sensitivity analysis, according to JNC 7. Factors associated with hypertension were assessed using logistic regression, with adjusted odds ratios (aORs) and 95% confidence intervals (CIs). The agreement between the two classifications was assessed using Cohen’s *κ* and McNemar’s test.

**Results:**

Among 3505 medical students, 1171 (33.4%) were classified as hypertensive according to the 2025 ACC/AHA criteria. In multivariable analysis, hypertension was independently associated with a family history of hypertension (aOR 1.31, 95% CI 1.09–1.59), higher BMI (aOR 1.02 per kg/m^2^, 95% CI 1.00–1.04), alcohol consumption (aOR 1.32, 95% CI 1.09–1.62) and physical inactivity (aOR 1.22, 95% CI 1.02–1.46). Using JNC 7, only 168 students (4.8%) were classified as hypertensive. In the sensitivity analysis, older age, higher academic level and a family history of hypertension were associated with JNC 7‐defined hypertension. The agreement between the two classifications was 71.4% (*κ* = 0.18, *p* < 0.001), while McNemar’s test indicated a significant difference in paired classifications (*χ* ^2^ = 1003.0, *p* < 0.001).

**Conclusion:**

Hypertension was highly prevalent among medical students when classified according to the 2025 ACC/AHA criteria, although the prevalence was substantially lower using JNC 7. The limited agreement between the two classifications highlights the considerable impact of diagnostic thresholds on the estimated burden of hypertension among young adults. Early identification of modifiable and familial factors may support targeted cardiovascular risk prevention in university populations.

## 1. Introduction

Hypertension (HTN) is a major modifiable risk factor for cardiovascular disease and premature mortality worldwide. It affects more than one billion adults globally, with a disproportionate burden in low‐ and middle‐income countries (LMICs), where detection, treatment and control remain suboptimal [1, 2]. Although HTN is traditionally regarded as a disease of middle and older age, elevated blood pressure (BP) is increasingly recognised among young adults and may contribute to the development of cardiovascular disease later in life [3–5].

The epidemiology of HTN in young adults is particularly relevant in sub‐Saharan Africa (SSA), where rapid urbanisation and changes in lifestyle have contributed to increasing exposure to cardiovascular risk factors, including excess body weight, physical inactivity and alcohol consumption [6–8]. Studies conducted in African populations have reported substantial heterogeneity in the prevalence of elevated BP and HTN, reflecting differences in population characteristics, measurement procedures and, importantly, the diagnostic thresholds applied [3, 5, 7]. Young adults attending universities may represent a particularly relevant population for early cardiovascular risk assessment because lifestyle patterns established during this period may persist into adulthood.

An important challenge in estimating the burden of HTN is the lack of consistency in BP classification across studies and over time. The Seventh Report of the Joint National Committee on Prevention, Detection, Evaluation and Treatment of High Blood Pressure (JNC 7) defined HTN as a systolic BP (SBP) ≥ 140 mmHg or a diastolic BP (DBP) ≥ 90 mmHg, while values of 120–139 mmHg systolic or 80–89 mmHg diastolic were classified as prehypertension (pre‐HTN) [9]. More recently, the 2025 American College of Cardiology/American Heart Association (ACC/AHA) Guideline for the Prevention, Detection, Evaluation and Management of High Blood Pressure in Adults retains the lower BP threshold introduced in the 2017 ACC/AHA guideline, defining Stage 1 HTN as SBP 130–139 mmHg or DBP 80–89 mmHg and Stage 2 HTN as SBP ≥ 140 mmHg or DBP ≥ 90 mmHg [10]. Consequently, the application of different classification systems to the same population can result in substantially different estimates of HTN prevalence and may also affect the factors identified as being associated with HTN.

This issue is particularly relevant in young populations, in whom relatively modest elevations in BP may be common while conventional JNC 7 thresholds identify fewer individuals as hypertensive. Comparisons between classification systems within the same population can therefore help distinguish differences attributable to the underlying epidemiology from those resulting from the diagnostic criteria themselves. Such comparisons are also important when interpreting and comparing findings from previous studies conducted in settings where JNC 7 or other BP thresholds were used.

In the Democratic Republic of the Congo (DRC), HTN is an increasingly recognised public health problem, with studies documenting a substantial burden among adults in both urban and rural settings [11–14]. However, evidence on BP levels and HTN among young adults, particularly university students, remains limited. Medical students constitute a relevant population in which to investigate early cardiovascular risk, given their exposure to demanding academic environments and potentially modifiable behavioural and lifestyle factors. Nevertheless, data on the prevalence and factors associated with HTN among medical students in the DRC remain scarce. Furthermore, differences in HTN prevalence across studies may partly reflect the diagnostic criteria used. The recent publication of the 2025 ACC/AHA guideline provides an opportunity to reassess the burden of HTN using contemporary classification criteria and to examine how prevalence estimates differ from those obtained using the historical JNC 7 definition in a large population of medical students in Lubumbashi.

Therefore, this study aimed to estimate the prevalence of HTN and identify factors associated with HTN among medical students at the University of Lubumbashi (UNILU), DRC. Within this population, students were classified according to their BP status, and those with HTN were compared with those without HTN to identify sociodemographic, behavioural, lifestyle and anthropometric factors associated with HTN. As a secondary objective, the same BP measurements were classified according to the JNC 7 and 2025 ACC/AHA criteria to assess the extent to which the choice of diagnostic threshold influences the estimated prevalence of HTN in this population.

## 2. Materials and Methods

### 2.1. Study Setting and Design

This cross‐sectional study was conducted from 1 July to 30 August 2023 among students of the Faculty of Medicine at the University of Lubumbashi (UNILU), in Lubumbashi, Haut‐Katanga Province, southeastern DRC. UNILU is one of the major public universities in the country, and its Faculty of Medicine enrols students across seven academic years. Lubumbashi is the capital and largest city of Haut‐Katanga Province and an important urban, academic and socioeconomic centre in the southeastern DRC. The city’s estimated population was 2,933,960 in 2024 [15].

### 2.2. Study Population and Sampling

The study population comprised students regularly enrolled in the Faculty of Medicine at UNILU during the 2022–2023 academic year. Students were eligible if they were aged ≥ 18 years and provided informed consent. Students with conditions potentially affecting BP or requiring treatments that could substantially influence BP were excluded. These conditions included severe chronic diseases such as kidney disease, diabetes mellitus, endocrine disorders, systemic lupus erythematosus and scleroderma, as well as conditions requiring ongoing treatment with systemic corticosteroids, nonsteroidal anti‐inflammatory drugs (NSAIDs), antidepressants, or hormonal contraceptives. Pregnant women were also excluded. Students aged < 18 years, enrolled in faculties other than medicine, those who declined participation, and those with incomplete study data were excluded.

The minimum sample size was calculated using the Cochrane formula for estimating a proportion in a cross‐sectional study: *n* = *Z* ^2^ *p* *q*/*d* ^2^, where *n* is the required sample size, *Z* is the standard normal deviate corresponding to a 95% confidence level (CI) (1.96), *p* is the expected prevalence of HTN, *q* = 1 − *p*, and *d* is the desired margin of error. Assuming an expected HTN prevalence of 33.6%, based on a previous study conducted among adults in Lubumbashi by Musung et al. [11], with a 5% margin of error, the minimum sample size was estimated at 348 participants. An additional 20% was allowed for potential nonresponse and incomplete data, giving a target sample size of approximately 418 participants.

All students enrolled in the seven academic years were subsequently approached during the study period using an inclusive recruitment strategy. Of the 4385 students enrolled in the Faculty of Medicine during the 2022–2023 academic year, 3505 participated in the study (79.93%). Among those not included, 663 declined participation, 154 were absent during data collection, 36 were pregnant, seven were aged < 18 years and 20 had incomplete data. The final analytical sample therefore comprised 3505 students, with participants recruited across all seven academic years (Figure 1).

**FIGURE 1:**
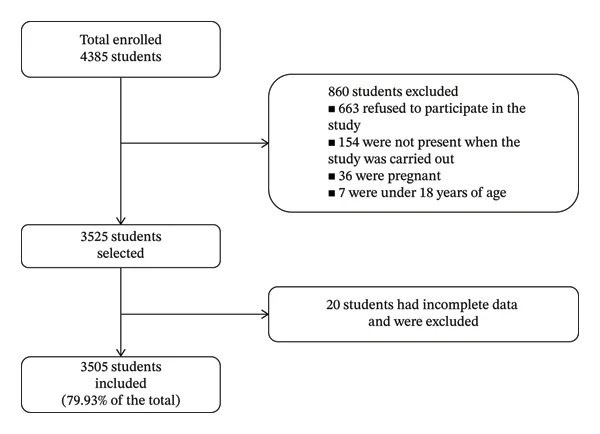
Flowchart illustrating participant selection.

### 2.3. Data Collection and Measurements

Data were collected by a team of 21 investigators comprising 10 final‐year medical students, 10 nurses and the principal investigator, a physician. Before data collection, all investigators received standardised training from the principal investigator on questionnaire administration, anthropometric measurements, BP assessment and the correct use of study equipment. A structured data collection form was developed to capture all study variables. An information and sensitisation campaign was conducted among students to explain the study objectives, procedures and importance.

Participants completed a standardised questionnaire and underwent a physical examination conducted by trained members of the study team. The questionnaire collected sociodemographic characteristics, including age, sex, marital status and academic level, as well as potential factors associated with HTN, including alcohol consumption, tobacco use, physical activity, dietary practices, family history of HTN and history of diabetes mellitus.

Before BP measurement, participants were instructed to avoid smoking, exercise and consumption of tea, coffee, or other caffeinated beverages for at least 30 min. BP was measured with an automated oscillometric sphygmomanometer (OMRON HEM‐7361T‐EBK, Kyoto, Japan) after the participant had been seated and resting for at least five minutes. Three consecutive measurements were obtained at intervals of at least 2 min. The mean of the second and third measurements was used to determine BP status.

Anthropometric measurements were performed by trained nurses according to standardised procedures. Body weight was measured to the nearest 0.1 kg using a calibrated digital scale (SECA 876, Hamburg, Germany), with participants barefoot and wearing light clothing. Height was measured to the nearest 0.1 cm using a rigid stadiometer, with participants standing barefoot in an upright position and maintaining the head in the appropriate anatomical position. Body mass index (BMI) was calculated as weight in kilograms divided by height in metres squared (kg/m^2^). Waist circumference was measured at the level of the iliac crest using a nonstretchable measuring tape, with the participant standing in a relaxed position at the end of a normal expiration. Hip circumference was measured at the level of the greatest circumference of the buttocks [16].

All BP and anthropometric measurement devices were calibrated with technical support from the Centre of Excellence in Laboratory and Health (CoHL), Kinshasa, DRC, to ensure measurement accuracy and consistency throughout the study.

### 2.4. Operational Definitions

Sociodemographic characteristics included age, sex, marital status and academic year. Age was analysed as a continuous variable (years). Sex was categorised as male or female and marital status as single or married. Academic level was grouped into 1st–3rd year and 4th–7th year according to the study curriculum.

Lifestyle characteristics included tobacco use, alcohol consumption, physical activity and dietary habits. Current tobacco use was defined as self‐reported current smoking of cigarettes or shisha. Participants were classified as tobacco users or non‐users. In this study population, all participants reporting tobacco use were daily smokers; therefore, smoking frequency was not analysed separately. Current alcohol consumption was defined as self‐reported alcohol use, whether occasional or regular and categorised as yes/no. Information on the quantity, frequency, or duration of alcohol consumption was not available.

Dietary variables included fruit and vegetable consumption. Dietary intake was assessed using self‐reported information collected through the study questionnaire. Fruit and vegetable consumption was classified as adequate when participants reported consuming at least five servings per day (approximately 400 g/day), in accordance with WHO recommendations for the prevention of noncommunicable diseases; otherwise, consumption was classified as suboptimal [17]. These variables were analysed as binary variables (suboptimal/adequate).

Physical activity was classified according to WHO recommendations. Participants were considered physically active if they reported at least 150 min of moderate‐intensity aerobic activity or 75 min of vigorous‐intensity activity per week, including activities related to work, transportation, or leisure; those who did not meet these thresholds were classified as physically inactive [17].

Anthropometric measurements included body weight, height, BMI and waist circumference. Weight and height were measured during the physical examination using calibrated equipment, with participants wearing light clothing and no shoes. BMI was calculated as weight in kilograms divided by height in metres squared (kg/m^2^). Both BMI and waist circumference were analysed as continuous variables in the regression models.

Family history of HTN was defined as self‐reported HTN in a first‐degree relative (mother, father, sister, or brother) diagnosed before the age of 60 years and was analysed as a binary variable (yes/no) [18].

NSAID use was assessed by self‐report and categorised as current use versus no use.

BP measurements collected in 2023 were subsequently classified according to two definitions (Table 1). The 2025 ACC/AHA guideline was used as the primary classification, distinguishing normal, elevated BP, Stage 1 HTN and Stage 2 HTN [10]. The JNC 7 definition [9] was used in a sensitivity analysis, distinguishing normal BP, pre‐HTN, Stage 1 HTN and Stage 2 HTN.

**TABLE 1:** Classification of blood pressure according to the 2025 ACC/AHA and JNC 7 definitions.

Classification	Category	SBP (mmHg)	DBP (mmHg)
2025 ACC/AHA	Normal blood pressure	< 120	and < 80
	Elevated blood pressure	120–129	and < 80
	Stage 1 hypertension	130–139	or 80–89
	Stage 2 hypertension	≥ 140	or ≥ 90

JNC 7	Normal blood pressure	< 120	and < 80
	Prehypertension	120–139	and/or 80–89
	Stage 1 hypertension	140–159	and/or 90–99
	Stage 2 hypertension	≥ 160	and/or ≥ 100

### 2.5. Statistical Analysis

Data were entered in Microsoft Excel 2019 and analysed using Stata Version 16.0 (StataCorp, College Station, TX, USA) [19]. Continuous variables were summarised as means ± standard deviation (SD), and categorical variables as frequencies and percentages. The distribution of continuous variables was assessed using the Shapiro–Wilk test. Depending on the distribution, continuous variables were compared between students with and without HTN using Student′s *t*‐test or the Mann–Whitney *U* test, as appropriate. Categorical variables were compared using Pearson’s *χ* ^2^ test or Fisher’s exact test when expected cell counts were small.

HTN prevalence was estimated according to the 2025 ACC/AHA classification and the JNC 7 definition. Under the 2025 ACC/AHA criteria, HTN was defined as SBP ≥ 130 mmHg or DBP ≥ 80 mmHg, whereas under JNC 7 it was defined as SBP ≥ 140 mmHg or DBP ≥ 90 mmHg. The prevalence of HTN was reported with corresponding 95% CIs. BP categories according to the 2025 ACC/AHA classification were also described as normal, elevated, Stage 1 HTN and Stage 2 HTN.

Agreement between the 2025 ACC/AHA and JNC 7 definitions was assessed using overall observed agreement and Cohen’s kappa (*κ*). Differences in paired classification between the two definitions were assessed using McNemar’s test.

Factors associated with HTN according to the 2025 ACC/AHA definition were first examined using univariable logistic regression, with unadjusted odds ratios (uORs), 95% CIs and *p*‐values reported. A multivariable logistic regression model was then fitted to identify factors independently associated with HTN. The model included age, sex, marital status, academic year, family history of HTN, BMI, waist circumference, alcohol consumption, physical activity, fruit consumption, vegetable consumption and NSAID use. Smoking was not included in the multivariable model because of the very small number of smokers and complete separation. Marital status was also excluded from the final model because of the very small number of married students and resulting separation. Adjusted odds ratios (aORs) with 95% CIs were reported.

As a sensitivity analysis, the same multivariable logistic regression approach was repeated using the JNC 7 definition of HTN as the outcome, allowing assessment of the robustness of the observed associations to the HTN definition used.

All statistical tests were two‐sided, and *p* < 0.05 was considered statistically significant.

## 3. Results

A total of 3505 medical students were included in the analysis, comprising 2142 males (61.1%) and 1363 females (38.9%) (Table 2). The overall mean age was 24.17 ± 3.50 years, with males being slightly older than females (24.36 ± 3.49 vs. 23.86 ± 3.49 years; *p* < 0.001). Males also had higher mean weight (66.46 ± 9.54 vs. 64.05 ± 10.56 kg; *p* < 0.001) and height (166.70 ± 10.73 vs. 163.10 ± 8.99 cm; *p* < 0.001), whereas mean BMI was similar between sexes (24.18 ± 4.57 vs. 24.26 ± 4.74 kg/m^2^; *p* = 0.590). Mean waist circumference was higher among males (75.38 ± 37.14 vs. 71.97 ± 14.37 cm; *p* = 0.001).

**TABLE 2:** Background characteristics of the study participants, by sex (*N* = 3505).

Variable	Female (*n* = 1363)	Male (*n* = 2142)	Total (*N* = 3505)	*p* value
Age (years), mean ± SD	23.86 ± 3.49	24.36 ± 3.49	24.17 ± 3.50	< 0.001
Weight (kg), mean ± SD	64.05 ± 10.56	66.46 ± 9.54	65.52 ± 10.02	< 0.001
Height (cm), mean ± SD	163.10 ± 8.99	166.70 ± 10.73	165.30 ± 10.24	< 0.001
Waist circumference (cm), mean ± SD	71.97 ± 14.37	75.38 ± 37.14	74.06 ± 30.43	0.001
BMI (kg/m^2^), mean ± SD	24.26 ± 4.74	24.18 ± 4.57	24.21 ± 4.64	0.590
Systolic blood pressure (mmHg), mean ± SD	116.46 ± 12.51	119.78 ± 10.30	118.49 ± 11.32	< 0.001
Diastolic blood pressure (mmHg), mean ± SD	72.13 ± 9.48	73.09 ± 8.56	72.71 ± 8.94	0.002
Heart rate (beats/min), mean ± SD	76.13 ± 10.18	73.87 ± 9.07	74.75 ± 9.58	< 0.001
Marital status, *n* (%)				0.276
Single	1348 (98.90)	2126 (99.25)	3474 (99.12)	
Married	15 (1.10)	16 (0.75)	31 (0.88)	
Academic year, *n* (%)				< 0.001
1st–3rd year	756 (55.47)	923 (43.09)	1679 (47.90)	
4th–7th year	607 (44.53)	1219 (56.91)	1826 (52.10)	
Family history of HTN, *n* (%)				0.194
No	1106 (81.14)	1775 (82.87)	2881 (82.20)	
Yes	257 (18.86)	367 (17.13)	624 (17.80)	
Alcohol consumption, *n* (%)				< 0.001
No	1203 (88.26)	1754 (81.89)	2957 (84.37)	
Yes	160 (11.74)	388 (18.11)	548 (15.63)	
Physical inactivity, *n* (%)				< 0.001
No	814 (59.72)	1128 (52.66)	1942 (55.41)	
Yes	549 (40.28)	1014 (47.34)	1563 (44.59)	
Fruit consumption, *n* (%)				0.063
Suboptimal	39 (2.86)	87 (4.06)	126 (3.59)	
Adequate	1324 (97.14)	2055 (95.94)	3379 (96.41)	
Vegetable consumption, *n* (%)				0.006
Suboptimal	42 (3.08)	36 (1.68)	78 (2.23)	
Adequate	1321 (96.92)	2106 (98.32)	3427 (97.77)	
Smoking, *n* (%)				0.788^∗^
No	1357 (99.56)	2134 (99.63)	3491 (99.60)	
Yes	6 (0.44)	8 (0.37)	14 (0.40)	
NSAID use, *n* (%)				< 0.001
No	974 (71.46)	1743 (81.37)	2717 (77.52)	
Yes	389 (28.54)	399 (18.63)	788 (22.48)	
History of diabetes mellitus, *n* (%)				1.000^∗^
No	1361 (99.85)	2139 (99.86)	3500 (99.86)	
Yes	2 (0.15)	3 (0.14)	5 (0.14)	

*Note:* Continuous variables are presented as mean ± SD and were compared between females and males using the independent‐samples *t*‐test. Categorical variables are presented as *n* (%) and percentages are calculated within sex (column percentages); groups were compared using Pearson’s *χ* ^2^ test. *p* < 0.05 was considered statistically significant. HTN, hypertension.

Abbreviations: BMI, body mass index; DBP, diastolic blood pressure; HR, heart rate; NSAID, nonsteroidal anti‐inflammatory drug; SBP, systolic blood pressure; SD, standard deviation.

^∗^Fisher’s exact test.

Regarding cardiovascular measurements, males had higher mean systolic (119.78 ± 10.30 vs. 116.46 ± 12.51 mmHg; *p* < 0.001) and DBP (73.09 ± 8.56 vs. 72.13 ± 9.48 mmHg; *p* = 0.002), whereas females had a higher mean heart rate (76.13 ± 10.18 vs. 73.87 ± 9.07 beats/min; *p* < 0.001).

Most participants were single (99.1%), and 52.1% were in the 4th–7th academic years. The distribution of academic year differed significantly by sex, with a higher proportion of females in the 1st–3rd years and males in the 4th–7th years (*p* < 0.001). A family history of HTN was reported by 17.8% of participants, with no significant difference by sex (*p* = 0.194).

Regarding lifestyle factors, 15.6% reported alcohol consumption, which was more frequent among males than females (18.1% vs. 11.7%; *p* < 0.001). Overall, 55.4% of participants were classified as physically inactive, with physical inactivity being more prevalent among females (59.7% vs. 52.7%; *p* < 0.001). Fruit consumption was reported by 96.4% of participants and did not differ significantly by sex (*p* = 0.063), whereas vegetable consumption was slightly more frequent among males (98.3% vs. 96.9%; *p* = 0.006). Tobacco use was rare (0.4%) and did not differ by sex (*p* = 0.760∗). NSAID use was reported by 22.5% of participants and was significantly more common among females than males (28.5% vs. 18.6%; *p* < 0.001). A history of diabetes mellitus was uncommon (0.14%) and showed no significant difference between sexes (*p* = 0.959).

Among the 3505 participants, the distribution of BP categories differed substantially according to the classification system applied (Table 3). Under the 2025 ACC/AHA criteria, 1460 participants (41.65%) had normal BP, 874 (24.94%) had elevated BP, 1003 (28.62%) were classified as having Stage 1 HTN, and 168 (4.79%) as having Stage 2 HTN. Accordingly, the overall prevalence of HTN, defined as SBP ≥ 130 mmHg or DBP ≥ 80 mmHg, was 33.41% (1171/3505; 95% CI: 31.86–34.99).

**TABLE 3:** Distribution of participants according to the 2025 ACC/AHA and JNC 7 blood pressure classifications.

Classification	Blood pressure category	Blood pressure threshold (mmHg)	*n*	%
2025 ACC/AHA	Normal	SBP < 120 and DBP < 80	1460	41.65
	Elevated	SBP 120–129 and DBP < 80	874	24.94
	Stage 1 hypertension	SBP 130–139 and/or DBP 80–89	1003	28.62
	Stage 2 hypertension	SBP ≥ 140 and/or DBP ≥ 90	168	4.79

JNC 7	Normal	SBP < 120 and DBP < 80	1460	41.65
	Prehypertension	SBP 120–139 and/or DBP 80–89	1877	53.55
	Stage 1 hypertension	SBP 140–159 and/or DBP 90–99	158	4.51
	Stage 2 hypertension	SBP ≥ 160 and/or DBP ≥ 100	10	0.29

*Note:* Categories within each blood pressure classification are mutually exclusive, and percentages are based on the total study population (*N* = 3505). For the 2025 ACC/AHA classification, hypertension comprises Stage 1 and Stage 2 hypertension (Stage 1 + Stage 2). For JNC 7, hypertension comprises Stage 1 and Stage 2 hypertension (Stage 1 + Stage 2). Thus, the corresponding overall prevalence of hypertension is 33.41% (1171/3505) according to the 2025 ACC/AHA classification and 4.80% (168/3505) according to JNC 7.

According to the JNC 7 definition, 1460 participants (41.65%) had normal BP, while 1877 (53.55%) were classified as having pre‐HTN. Stage 1 HTN was observed in 158 participants (4.51%), and Stage 2 HTN in 10 (0.29%). Thus, the overall prevalence of HTN according to JNC 7, defined as SBP ≥ 140 mmHg or DBP ≥ 90 mmHg, was 4.79% (168/3505; 95% CI: 4.13–5.55). Overall, the prevalence of HTN was therefore substantially higher under the 2025 ACC/AHA criteria than under the JNC 7 definition (33.41% vs. 4.79%). This marked difference primarily reflects the lower BP thresholds used to define HTN in the 2025 ACC/AHA criteria compared with the JNC 7 definition.

An agreement of 71.38% was observed between the 2025 ACC/AHA and JNC 7 definitions, with an expected agreement of 65.00%. However, the agreement beyond that expected by chance was poor (Cohen’s *κ* = 0.1824, *p* < 0.001). Among the 1171 students classified as having HTN according to the 2025 ACC/AHA criteria, 1003 (85.65%) were not classified as having HTN according to JNC 7, whereas only 168 (14.35%) were classified as having HTN by both definitions. No student was classified as having HTN according to JNC 7 but not according to the 2025 ACC/AHA criteria. McNemar’s test confirmed a significant difference in the classification of students between the two definitions (*χ* ^2^ = 1003.0, *p* < 0.001) (Table 4).

**TABLE 4:** Agreement between the 2025 ACC/AHA and JNC 7 definitions of hypertension among university students (*n* = 3505).

	JNC 7: no hypertension	JNC 7: hypertension	Total
ACC/AHA 2025: No hypertension	2334 (66.59%)	0 (0.00%)	2334 (66.59%)
ACC/AHA 2025: hypertension	1003 (28.62%)	168 (4.79%)	1171 (33.41%)
Total	3337 (95.21%)	168 (4.79%)	3505 (100.00%)

*Note:* Percentages are based on the total study population (*N* = 3505). Overall agreement between the 2025 ACC/AHA and JNC 7 definitions was 71.38%, compared with 65.00% expected by chance. Cohen’s *κ* was 0.1824 (*p* < 0.001), indicating low agreement beyond chance. McNemar’s test showed a significant difference in paired hypertension classification between the two definitions (*χ* ^2^(1) = 1003.00, *p* < 0.001).

According to the 2025 ACC/AHA criteria, students with HTN were slightly younger than those without HTN (23.91 ± 3.62 vs. 24.29 ± 3.43 years; *p* = 0.002). The proportion of students in their first to third year of study was higher among those with HTN (50.38% vs. 46.66%; *p* = 0.037), as was the prevalence of a family history of HTN (21.43% vs. 15.98%; *p* < 0.001). BMI did not differ significantly between the groups (*p* = 0.190), whereas abdominal obesity was paradoxically less frequent among students with HTN (6.58% vs. 9.98%; *p* = 0.001). Their mean heart rate was also slightly higher (76.13 ± 10.30 vs. 74.05 ± 9.12 beats/min; *p* < 0.001). Regarding behavioural factors, alcohol consumption (19.64% vs. 13.62%), physical inactivity (48.68% vs. 42.54%), and NSAID use (25.70% vs. 20.87%) were more frequent among students with HTN (all *p* ≤ 0.001). Sex, marital status and consumption of fruits and vegetables were not significantly associated with HTN status. Smoking was very rare in this population and was not reported by any student with HTN, limiting the interpretation of this association (Table 5).

**TABLE 5:** Characteristics of students according to hypertension status defined by the 2025 ACC/AHA criteria.

Variable	No hypertension (*n* = 2334)	Hypertension (*n* = 1171)	*p* value
Age (years), mean ± SD	24.29 ± 3.43	23.91 ± 3.62	0.002
Sex, *n* (%)			0.135
Male	1406 (60.24)	736 (62.85)	
Female	928 (39.76)	435 (37.15)	
Marital status, *n* (%)			0.891
Single	2313 (99.10)	1161 (99.15)	
Married	21 (0.90)	10 (0.85)	
Academic year, *n* (%)			0.037
1st–3rd year	1089 (46.66)	590 (50.38)	
4th–7th year	1245 (53.34)	581 (49.62)	
Family history of hypertension, *n* (%)			< 0.001
No	1961 (84.02)	920 (78.57)	
Yes	373 (15.98)	251 (21.43)	
BMI (kg/m^2^), mean ± SD	24.14 ± 4.74	24.36 ± 4.43	0.190
Waist circumference (cm), mean ± SD	74.51 ± 36.05	73.15 ± 13.39	0.213
Heart rate (beats/min), mean ± SD	74.05 ± 9.12	76.13 ± 10.30	< 0.001
Alcohol consumption, *n* (%)			< 0.001
No	2016 (86.39)	941 (80.36)	
Yes	318 (13.62)	230 (19.64)	
Physical inactivity, *n* (%)			0.001
Yes	993 (42.54)	570 (48.68)	
No	1341 (57.46)	601 (51.32)	
Fruit consumption, *n* (%)			0.453
Suboptimal	80 (3.43)	46 (3.93)	
Adequate	2254 (96.57)	1125 (96.07)	
Vegetable consumption, *n* (%)			0.087
Suboptimal	59 (2.53)	19 (1.62)	
Adequate	2275 (97.47)	1152 (98.38)	
Smoking, *n* (%)			0.004^∗^
No	2320 (99.40)	1171 (100.00)	
Yes	14 (0.60)	0 (0.00)	
NSAID use, *n* (%)			0.001
No	1847 (79.13)	870 (74.30)	
Yes	487 (20.87)	301 (25.70)	

*Note:* Continuous variables are presented as mean ± SD; categorical variables as *n* (%). *p*‐values were obtained using Student’s *t*‐test for continuous variables and Pearson’s *χ* ^2^ test for categorical variables, except for smoking, for which Fisher’s exact test was used.

^∗^Fisher’s exact test.

In the bivariate analysis, age, year of study, family history of HTN, alcohol consumption, physical activity and NSAID use were associated with HTN. After multivariable adjustment, family history of HTN (aOR = 1.31; 95% CI: 1.09–1.59; *p* = 0.005), BMI (aOR = 1.02 per kg/m^2^; 95% CI: 1.003–1.036; *p* = 0.018), alcohol consumption (aOR = 1.32; 95% CI: 1.09–1.62; *p* = 0.006) and physical inactivity (aOR = 1.22; 95% CI: 1.02–1.46; *p* = 0.031) remained significantly associated with HTN. The association with age became borderline after adjustment (aOR = 0.98; 95% CI: 0.95–1.00; *p* = 0.055). Sex, marital status, year of study, waist circumference, fruit and vegetable consumption and NSAID use were no longer significantly associated with HTN in the multivariable model (Table 6).

**TABLE 6:** Factors associated with hypertension among students according to the ACC/AHA 2025 criteria.

Variable	Unadjusted OR	95% CI	*p* value	Adjusted OR	95% CI	*p* value
Age, per 1‐year increase	0.969	0.950–0.989	0.002	0.976	0.952–1.001	0.055
Male vs. female	1.117	0.966–1.291	0.135	1.108	0.953–1.288	0.183
Married vs. single	0.949	0.445–2.021	0.891	1.010	0.471–2.168	0.979
4th–7th year vs. 1st–3rd year	0.861	0.748–0.991	0.037	1.108	0.898–1.365	0.339
Family history of hypertension: yes vs. no	1.434	1.200–1.714	< 0.001	1.313	1.086–1.588	0.005
BMI, per 1 kg/m^2^ increase	1.010	0.995–1.025	0.190	1.020	1.003–1.036	0.018
Waist circumference, per 1 cm increase	0.998	0.995–1.001	0.225	0.998	0.995–1.001	0.109
Alcohol consumption: yes vs. no	1.550	1.286–1.867	< 0.001	1.324	1.086–1.615	0.006
Physical inactivity: yes vs. no	1.281	1.112–1.475	0.001	1.217	1.018–1.456	0.031
Fruit consumption: adequate vs. suboptimal	0.868	0.600–1.256	0.453	0.942	0.637–1.392	0.763
Vegetable consumption: adequate vs. suboptimal	1.572	0.933–2.650	0.089	1.675	0.977–2.871	0.061
NSAID use: yes vs. no	1.312	1.113–1.547	0.001	1.190	0.997–1.419	0.054

*Note:* Adjusted ORs were obtained from a multivariable logistic regression model including age, sex, marital status, academic year, family history of hypertension, BMI, waist circumference, alcohol consumption, physical activity, fruit consumption, vegetable consumption and NSAID use. Smoking was not included because of perfect prediction/separation (no hypertensive participant reported smoking).

Abbreviations: CI, confidence interval; NE, not estimable; NSAID, nonsteroidal anti‐inflammatory drug; OR, odds ratio.

The multivariable analysis based on the JNC 7 definition showed that age, year of study, and family history of HTN were significantly associated with HTN (Table 7). Each additional year of age was associated with an 11.3% increase in the odds of HTN (aOR = 1.113; 95% CI: 1.059–1.171; *p* < 0.001). Conversely, students in their 4th–7th year of study had significantly lower odds of HTN than those in their 1st–3rd year (aOR = 0.370; 95% CI: 0.238–0.576; *p* < 0.001). A family history of HTN was associated with approximately 1.6‐fold higher odds of HTN (aOR = 1.646; 95% CI: 1.113–2.434; *p* = 0.013). In contrast, no statistically significant associations were observed with sex, BMI, waist circumference, alcohol consumption, physical inactivity, fruit or vegetable consumption, or NSAID use (*p* > 0.05). These findings represent a sensitivity analysis using the JNC 7 definition and indicate that the factors associated with HTN differed partly from those identified using the 2025 ACC/AHA criteria, highlighting the influence of the BP classification criteria on the observed associations.

**TABLE 7:** Factors associated with hypertension according to JNC 7 criteria: sensitivity analysis.

Variable	Adjusted OR	95% CI	*p* value
Age, per 1‐year increase	1.113	1.059–1.171	< 0.001
Male vs. female	0.874	0.631–1.212	0.420
4th–7th year vs. 1st–3rd year	0.370	0.238–0.576	< 0.001
Family history of hypertension	1.646	1.113–2.434	0.013
BMI, per 1 kg/m^2^ increase	1.001	0.963–1.040	0.965
Waist circumference, per 1 cm increase	1.001	0.997–1.005	0.536
Alcohol consumption	1.246	0.830–1.871	0.288
Physical inactivity suboptimal	1.022	0.697–1.498	0.912
Fruit consumption suboptimal	0.948	0.452–1.989	0.888
Vegetable consumption	1.724	0.520–5.723	0.373
NSAID use	1.182	0.816–1.714	0.376

*Note:* Adjusted odds ratios were estimated using multivariable logistic regression. The model included age, sex, academic year, family history of hypertension, BMI, waist circumference, alcohol consumption, physical activity, fruit consumption, vegetable/legume consumption and NSAID use. Marital status was excluded because of quasi‐complete/perfect separation resulting from the very small number of married students. Smoking was also excluded because of perfect prediction (no smokers were hypertensive).

Abbreviations: CI, confidence interval; NE, not estimable; NSAID, nonsteroidal anti‐inflammatory drug; OR, odds ratio.

## 4. Discussion

HTN is increasingly recognised as an important cardiovascular risk factor among young adults, including university students. However, estimates of its prevalence are highly dependent on the BP thresholds used to define the condition. In this large cross‐sectional study of 3505 medical students in Lubumbashi, the estimated prevalence of HTN differed markedly according to the classification applied. Using the 2025 ACC/AHA criteria, 33.4% of participants were classified as having HTN, whereas only 4.8% met the definition of HTN according to the JNC 7 criteria. This nearly sevenfold difference illustrates the substantial influence of diagnostic thresholds on estimates of HTN burden in young adult populations.

To our knowledge, this is among the first studies in the DRC to directly compare the impact of the 2025 ACC/AHA and JNC 7 classifications on the estimated prevalence of HTN among university students. Under the 2025 ACC/AHA criteria, only 41.7% of participants had normal BP, while 24.9% had elevated BP, 28.6% had Stage 1 HTN, and 4.8% had Stage 2 HTN. In contrast, under JNC 7, more than half of the students (53.55%) were classified as having pre‐HTN, whereas only 4.8% were classified as hypertensive. These findings indicate that a substantial proportion of young adults previously classified as having pre‐HTN under JNC 7 are reclassified as having HTN, particularly Stage 1 HTN, when lower 2025 ACC/AHA diagnostic thresholds are applied.

The prevalence of HTN observed using the JNC 7 definition was comparable with estimates previously reported among university students in several African settings. For example, prevalences of approximately 4%–8% have been reported among university students in Côte d’Ivoire, Kisangani and Kinshasa [20–22]. Similarly, studies conducted in Ghana and Asian university populations have reported relatively low prevalences of HTN, ranging from 1.4% to 4.3%, when traditional thresholds of SBP ≥ 140 mmHg and/or DBP ≥ 90 mmHg were used [18, 23, 24]. However, substantially higher prevalences, ranging from 12.7% to 14.0%, have been reported among university students in other African settings, including Cameroon and Uganda [25, 26]. Differences between studies may partly reflect variations in age distribution, sex composition, body weight, lifestyle characteristics, BP measurement procedures and, importantly, the diagnostic criteria used.

The considerably higher prevalence observed with the 2025 ACC/AHA criteria should therefore not necessarily be interpreted as evidence of a sudden or disproportionate increase in clinically established HTN among medical students. Rather, it largely reflects the consequences of lowering the diagnostic threshold from 140/90 mmHg to 130/80 mmHg. In the present study, 1003 students (28.6%) were classified as having Stage 1 HTN according to the 2025 ACC/AHA criteria, a category that largely overlaps with the pre‐HTN range under the JNC 7 classification. This reclassification substantially contributed to the difference in HTN prevalence observed between the two diagnostic systems. This finding is consistent with evidence from other populations showing that implementation of lower BP thresholds substantially increases the estimated prevalence of HTN, particularly among younger adults who frequently have mildly elevated BP.

This issue is particularly relevant in young populations. Although young adults with Stage 1 HTN generally have a lower absolute short‐term cardiovascular risk than older adults with established HTN, elevated BP during early adulthood may persist or progress over time. BP tends to track from adolescence and young adulthood into later life, and individuals with elevated BP may subsequently develop sustained HTN and a higher cardiovascular risk. Consequently, the identification of young adults with Stage 1 HTN may provide an opportunity for early intervention, particularly through lifestyle modification and periodic BP monitoring.

In the present study, a family history of HTN was independently associated with HTN defined according to the ACC/AHA criteria (aOR 1.31, 95% CI 1.09–1.59). This finding is biologically plausible and consistent with previous studies reporting an increased risk of elevated BP among individuals with a family history of HTN [27, 28]. A family history may reflect both inherited susceptibility and shared environmental or behavioural exposures. Genetic factors involved in vascular regulation, renal sodium handling, sympathetic nervous system activity and the renin–angiotensin–aldosterone system may contribute to individual susceptibility to elevated BP [29]. In addition, family members may share dietary habits, levels of physical activity and other behavioural characteristics that influence cardiovascular risk. The association observed in this study therefore supports the potential value of incorporating family history into cardiovascular risk assessment among young adults.

Higher BMI was also independently associated with HTN, with each one‐unit increase in BMI associated with a 2% increase in the odds of 2025 ACC/AHA‐defined HTN. Although the magnitude of this association was modest, it reinforces the importance of body weight as a cardiovascular risk factor even within a relatively young and predominantly healthy university population. Previous studies among students and young adults have consistently reported associations between excess body weight and HTN [23, 30–34].

Several biological mechanisms may explain the relationship between increased adiposity and HTN. Excess adipose tissue may contribute to sympathetic nervous system activation, insulin resistance, endothelial dysfunction, oxidative stress and activation of the renin–angiotensin–aldosterone system. Obesity‐related changes in renal haemodynamics and sodium handling may further contribute to the development of HTN [35–37]. Although the average BMI in this study population was within the normal range, the observed association suggests that even relatively small increases in body weight may be relevant to BP regulation among young adults.

Alcohol consumption was another independent factor associated with 2025 ACC/AHA‐defined HTN. Students who reported alcohol consumption had approximately 32% higher odds of HTN than nonconsumers. This finding is consistent with previous evidence linking alcohol consumption to HTN [34, 38, 39], although some studies among university students have reported no statistically significant association [23, 26]. The effect of alcohol on BP may vary according to the frequency and quantity of consumption, patterns of drinking and individual susceptibility. Alcohol may contribute to increased sympathetic activity, vascular dysfunction and neurohormonal changes that promote BP elevation. The observed association nevertheless has important preventive implications in university settings. Young adulthood is a period during which behavioural patterns may become established and persist into later life. Health promotion interventions targeting alcohol consumption may therefore have potential benefits beyond the immediate prevention of HTN, including broader cardiovascular and noncommunicable disease prevention.

Physical inactivity was also independently associated with HTN according to the 2025 ACC/AHA classification. Students who did not engage in physical activity had 22% higher odds of HTN compared with those reporting physical activity. This finding supports the established role of regular physical activity in BP regulation and cardiovascular health. Exercise may reduce BP through improvements in endothelial function, vascular compliance, autonomic regulation and insulin sensitivity, as well as reductions in peripheral vascular resistance [40, 41]. The association between physical inactivity and HTN is particularly important in university populations, where academic demands may promote sedentary behaviour. Medical students may experience prolonged periods of studying, clinical training and other academic activities that limit opportunities for regular exercise. University‐based interventions promoting physical activity could therefore represent a practical approach to cardiovascular risk prevention. Such interventions could include increased access to recreational facilities, structured physical activity programmes and health promotion initiatives integrated into student life.

The marked difference in HTN prevalence between the two classifications was further reflected in the limited agreement between the 2025 ACC/AHA and JNC 7 definitions. Although the overall agreement between classifications was 71.4%, Cohen’s *κ* was only 0.18, indicating poor agreement beyond what is expected by chance. McNemar’s test also demonstrated a highly significant difference between the classifications (*χ* ^2^ = 1003.0, *p* < 0.001). These findings demonstrate that the two systems do not identify the same group of students as hypertensive. The apparent discrepancy between the relatively high percentage agreement and the low *κ* coefficient deserves consideration. In populations where one outcome category is substantially more common than another, overall agreement may be influenced by concordant classification of the dominant category. Cohen’s *κ* adjusts for agreement expected by chance and may therefore provide a more informative assessment of concordance. In the present study, the low *κ* value indicates that the apparent overall agreement masks substantial reclassification when the 2025 ACC/AHA thresholds are applied. This finding has important epidemiological implications. Comparisons of HTN prevalence across studies should account for the classification system used, particularly when comparing studies conducted before and after the introduction of lower 2025 ACC/AHA thresholds. Failure to distinguish between diagnostic criteria may lead to misleading conclusions regarding temporal trends or geographical differences in HTN prevalence. A population may appear to have a substantially higher burden of HTN simply because a lower diagnostic threshold has been adopted.

From a clinical and public health perspective, the lower ACC/AHA threshold also raises an important question regarding the management of young adults identified with Stage 1 HTN. The identification of a larger proportion of young individuals with elevated BP does not imply that all require pharmacological treatment. Rather, for many young adults without established cardiovascular disease or high overall cardiovascular risk, initial management may emphasise confirmation of elevated BP, lifestyle modification and regular follow‐up. Nevertheless, early identification may provide an opportunity to prevent the progression from mildly elevated BP to sustained HTN.

The results of the sensitivity analysis using the JNC 7 definition also provide useful insights. Under this more conservative definition, HTN was associated with older age, higher academic level and a family history of HTN. These findings should, however, be interpreted cautiously because only 168 participants met the JNC 7 definition of HTN. The smaller number of cases reduces statistical precision and may partly explain differences in the pattern of associated factors compared with the 2025 ACC/AHA definition. More importantly, these differences reinforce the conclusion that the choice of diagnostic threshold may influence not only estimates of disease prevalence but also the apparent profile of factors associated with HTN.

### 4.1. Strengths and Limitations

This study has several strengths. First, it included a large sample of 3505 medical students, providing substantial statistical power for the estimation of BP categories and associated factors. Second, BP and anthropometric measurements were obtained using standardised procedures. Third, the simultaneous application of the 2025 ACC/AHA and JNC 7 classifications allowed a direct assessment of the impact of different diagnostic thresholds within the same population, thereby avoiding many of the limitations associated with comparisons between separate studies. Finally, the assessment of agreement using both Cohen’s *κ* and McNemar’s test provided complementary information regarding concordance and systematic differences between classifications.

Several limitations should nevertheless be acknowledged. First, the cross‐sectional design precludes causal inference. The observed associations between family history, BMI, alcohol consumption, physical inactivity and BP cannot establish temporal or causal relationships. Second, behavioural variables were self‐reported and may therefore be affected by recall bias or social desirability bias, particularly for alcohol consumption, smoking and physical activity. Third, the very low prevalence of smoking and diabetes mellitus in this young population limited the ability to evaluate their associations with HTN. Fourth, the study was conducted among medical students at a single university. Participants may differ from the general population of young adults in Lubumbashi with regard to education, socioeconomic circumstances, health knowledge and health‐related behaviours. The findings may therefore not be directly generalisable to all young adults in the DRC. Finally, BP classification based on measurements obtained during a cross‐sectional survey may overestimate the prevalence of sustained HTN because the diagnosis of HTN in clinical practice generally requires confirmation through repeated measurements on separate occasions or out‐of‐office BP assessment.

Despite these limitations, the study provides important evidence regarding the burden of HTN among young adults in the DRC and demonstrates the substantial impact of diagnostic thresholds on HTN prevalence estimates. The findings support the need for regular BP screening and cardiovascular risk assessment in university populations, particularly among students with a family history of HTN, higher BMI, alcohol consumption or low levels of physical activity.

## 5. Conclusion

In this large sample of medical students in Lubumbashi, the estimated prevalence of HTN varied substantially according to the diagnostic classification used. HTN affected one‐third of participants when defined according to the 2025 ACC/AHA criteria, compared with fewer than one in 20 when the JNC 7 threshold was applied. The lower 2025 ACC/AHA threshold resulted in the reclassification of a substantial proportion of students who would previously have been considered pre‐HTN.

Family history of HTN, higher BMI, alcohol consumption and physical inactivity were independently associated with 2025 ACC/AHA‐defined HTN. The poor agreement between the 2025 ACC/AHA and JNC 7 classifications further demonstrates that the choice of diagnostic threshold substantially influences both estimates of HTN burden and the identification of individuals considered at risk.

These findings highlight the importance of clearly specifying the classification used when reporting HTN prevalence, particularly in young adult populations. They also support early BP screening and lifestyle‐based cardiovascular prevention within university settings. Longitudinal studies are needed to determine whether students classified with Stage 1 HTN according to the 2025 ACC/AHA criteria progress to sustained HTN and experience an increased long‐term cardiovascular risk.

**Nomenclature**
2025 ACC/AHA: 2025 American College of Cardiology/American Heart Association
95% CI: 95% confidence intervals
aOR: Adjusted odds ratio
BMI: Body mass index
BP: Blood pressure
CoHL: Congolese Hypertension League
DBP: Diastolic blood pressure
DRC: Democratic Republic of the Congo
HTN: Hypertension
JNC 7: Joint National Committee 7
LMICs: Low‐ and middle‐income countries
NCDs: Noncommunicable diseases
NSAIDs: Nonsteroidal anti‐inflammatory drugs
Pre‐HTN: Prehypertension
SBP: Systolic blood pressure
SD: Standard deviation
SSA: Sub‐Saharan Africa
UNILU: University of Lubumbashi
WHO: World Health Organization

## Author Contributions

Jacques Mbaz Musung, Olivier Mukuku, Emmanuel Kiyana Muyumba and Phillipe Bianga Katchunga conceptualised the study and designed the methodology; Jacques Mbaz Musung, Olivier Mukuku and Didier Lez Malamba developed the software; Jacques Mbaz Musung, Emmanuel Kiyana Muyumba and Placide Kambola Kakoma conducted the validation. Olivier Mukuku, Marcellin Bugeme, Yves Idi Isango, Berthe Mwad Kon Kamalo and Placide Kambola Kakoma performed formal analysis; Jacques Mbaz Musung, Orly Muyumba Ngungwa, Harvey Kabulo Kapya and Berthe Mwad Kon Kamalo collected the data; Jacques Mbaz Musung, Orly Muyumba Ngungwa, Yves Idi Isango, Marcellin Bugeme and Didier Lez Malamba prepared the original draft of the manuscript; Olivier Mukuku, Emmanuel Kiyana Muyumba and Phillipe Bianga Katchunga provided supervision and contributed to the review and editing.

## Funding

This research received no external funding.

## Disclosure

All authors reviewed and approved the final version of the manuscript for publication.

## Ethics Statement

The study was approved by the University of Lubumbashi’s Medical Ethics Committee (approval number: UNILU/CEM/105/2022). It was conducted in accordance with the ethical principles set out in the Declaration of Helsinki for research involving human beings, as well as the ethical guidelines for research in the DRC. Written informed consent was obtained from each participant prior to their inclusion in the study, ensuring their understanding of the objectives, procedures and their rights, including their right to withdraw at any time without impacting the care they received. Confidentiality and anonymity in data management and analysis were scrupulously respected. All information collected has been anonymised to ensure that participants cannot be identified, and the data will only be used for this research and awareness‐raising purposes.

## Consent

Please see the Ethics Statement.

## Conflicts of Interest

The authors declare no conflicts of interest.

## Data Availability

The datasets used and/or analysed during the current study are available from the corresponding author on reasonable request.
